# Cross-generational comparison of reproductive success in recently caught strains of *Drosophila melanogaster*

**DOI:** 10.1186/s12862-017-0887-1

**Published:** 2017-02-06

**Authors:** Trinh T. X. Nguyen, Amanda J. Moehring

**Affiliations:** Department of Biology, Western University, London, ON N6A 5B7 Canada

**Keywords:** Lifetime reproductive success, LRS, Parent-offspring conflict, Cockerham & Weir biomodel

## Abstract

**Background:**

Males and females often have opposing strategies for increasing fitness. Males that out-compete others will acquire more mating opportunities and thus have higher lifetime reproductive success. Females that mate with a high quality male receive either direct benefits through productivity or acquisition of additional resources or indirect benefits through the increased fitness of offspring. These components may be in conflict: factors that increase offspring fitness may decrease a female’s productivity, and alleles that are beneficial in one sex may be detrimental in the opposite sex. Here, we use a multigenerational study with recently caught strains of *Drosophila melanogaster* to examine the relationship between parental, male offspring, and female offspring fitness when fitness is measured in a basal non-competitive environment.

**Results:**

We find synergy between parental and offspring lifetime reproductive success, indicating a lack of parent-offspring conflict, and a synergy between son and daughter reproductive success, indicating a lack of intersexual conflict. Interestingly, inbreeding significantly reduced the lifetime reproductive success of daughters, but did not have a significant effect on short-term productivity measures of daughters, sons or parents.

**Conclusions:**

In wild-caught flies, there appears to be no parent-offspring conflict or intersexual conflict for loci influencing offspring production in a anon-competitive environment. Further, there may not be a biologically relevant selection pressure for avoidance of inbreeding depression in wild-type individuals of this short-lived species.

**Electronic supplementary material:**

The online version of this article (doi:10.1186/s12862-017-0887-1) contains supplementary material, which is available to authorized users.

## Background

One of the most important aspects in evolution is an animal’s ability to reproduce, making reproductive success a vital measure of fitness. Males and females often have differing reproductive strategies to increase their reproductive success [1]. Males typically increase their fitness by competing and acquiring as many mating opportunities as possible. Variation in reproductive success is thus usually larger for males than it is for females, since some males may not achieve any matings while others achieve multiple matings [1]. In contrast, females are usually mated, and tend to have lower variation in reproductive success than males. While there may be some advantages to females for polyandry [2–4], there are also costs [5–9], and females may instead increase their fitness by mating selectively.

Females can increase their fitness through the direct benefits of increased offspring production and the indirect genetic benefits of increased offspring quality [10–13]. There are a variety of ways that a female may potentially increase the fitness of the resulting offspring. Females may choose mates based on traits that signal good genes, resulting in superior growth, fecundity, or survival of the offspring [1, 14]. The relationship between female mate preferences and the increased fitness of the resulting offspring has been shown in a variety of organisms, including pronghorn [15], poison frogs [16], and within a meta-analysis [17], among others. These studies indicate that females preferentially mate with males who signal honest indicators of good genes in order to confer a fitness advantage to their offspring (but see [18, 19]).

Females can also acquire non-additive genetic benefits by mating with males with whom they are genetically compatible [20]. Females can have a preference for unrelated males to avoid inbreeding, which can result in decreased offspring fitness due to increased homozygosity and expression of deleterious mutations, and a decrease in heterozygote advantage (e.g., [21–23]. but see [24]). For example, a well-documented system of genetic compatibility involves the major histocompatibility complex (MHC) genes, which are highly polymorphic loci that influence immune function by promoting immune response and resistance to infections and diseases [25, 26]. Females of many organisms have a preference for males with dissimilar MHC alleles [25–27], producing offspring with a better immune response that can recognize more pathogens, and thus increasing offspring fitness. These studies emphasize the importance of sexual selection and mate choice on offspring fitness through indirect additive and non-additive genetic benefits.

How these benefits manifest may involve cross-generational trade-offs, whereby a female can suffer decreased offspring production but produce higher quality offspring [28], and/or sex-specific trade-offs in the fitness of the resulting male and female offspring due to differential investment or sexual conflict [29]. Since most genes are expressed in both sexes, but the sexes can experience different selection pressures, there can be intersexual genetic conflict whereby alleles can be beneficial in one sex but harmful to the other [30, 31]. In some cases, sexual conflict is extreme enough to cause a decrease in lifespan and even death [9, 32, 33].

Together, these studies provide extensive evidence for the ability of a female to mate selectively based on a male’s representative phenotype in order to increase her own productivity and the fitness of the resulting offspring, but this fitness benefit may only apply to one sex of offspring. While a handful of studies have examined the more extreme effect of inbreeding on the fitness of parents and each sex of resulting offspring (e.g., [34–36]), very few studies have examined the general relationship between parental fitness and the fitness of the resulting male and female offspring [37], and most studies use lab-adapted rather than recently wild-caught flies, and thus a number of questions remain poorly understood. Here, we tested multiple aspects of the relationship between parental fitness and offspring fitness using wild-type strains of flies. Our first aim was to identify the genetic relationship between parental and offspring fitness. We obtained reproductive success measurements in *D. melanogaster* for parentals and all F_1_ individuals (both sons and daughters) from a full factorial diallel cross using recently-collected isofemale lines. The relationship between parental productivity and the productivity of offspring will determine whether the genes that confer increased productivity in parents are heritable and beneficial to the offspring of either or both sexes. The relationship between the productivity of the male and female offspring will determine if there is a trade-off in fitness due to sexual conflict.

Our second aim was to identify the genetic and parental effects contributing to variation in reproductive success. We used multiple simple regressions to analyze additive, paternal and maternal genetic effects, and then used the more complex Cockerham and Weir Biomodel [38], to partition the variance in productivity into additive, non-additive, maternal and paternal genetic effects. The relationship between the additive, maternal and paternal effects and the fitness of sons and daughters will determine if there is a particular contribution of genes from either the maternal or paternal genome that benefits sons and/or daughters.

Lastly, we identified the effects of inbreeding across generations and between males and females to determine if there were effects of inbreeding on lifetime reproductive success. This will determine if one sex is not more susceptible to the detrimental effects of inbreeding than the other.

## Methods

### Isofemale lines

Isofemale lines of *Drosophila melanogaster* were started from individual females collected from the wild in Sudbury, Ontario Canada in July 2011, generously provided by T. Merritt. Rearing methods are the same as in [39]. The lines were scored for the current experiment between April and October, 2012. Isofemale populations were reared and assayed on standard cornmeal agar and maintained at 24 °C and 75% RH on a 14 h light: 10 h dark cycle. A total of 10 isofemale lines were used in this experiment. Each line was kept with non-overlapping generations as a population of approximately 500 flies distributed among vials that were intermittently intermixed.

### Diallel cross and fitness measures

Diallel crossing methods are similar to those of [39]. Ten isofemale lines were used in a full diallel cross, mating females and males in all 100 possible line combinations. Male and female virgins were collected upon eclosion, aged 4–6 days, and mated. Mated pairs were kept together, allowing for remating. After 7 days, the male and female were transferred to a new vial. All offspring that eclosed from this first vial were counted (7 day productivity).

Males and females continued to be transferred to new vials every 7 days until no more offspring were produced. Mated pairs were checked daily and dead males were replaced with a male of similar age and strain. Vials were checked daily and counted for number of eclosing adult offspring. Vials were counted for 16–17 days after the last egg was laid or the female died, ensuring enough time for all larvae to emerge, providing a measure of total lifetime reproductive success (LRS). A total of 4 replicates of the complete 10x10 diallel cross were performed (400 pairings total). All crosses were represented and scored simultaneously within each replicate to control for environmental effects.

To measure F_1_ productivity, four F_1_ males (sons) and four F_1_ females (daughters) were taken from the first 7 days of offspring production for each of the four replicates of the 100 diallel crosses (for a total of 1600 F_1_ males and 1600 F_1_ females). As above, all offspring were represented and scored simultaneously within each replicate to control for environmental effects. Each F_1_ focal son was paired in a vial with a single standard female, and each F_1_ female was paired with a single standard male, allowing for remating. Standard females and males used in F_1_ mating pairs are from an outbred (synthetic) population made from combining two virgin males and two virgin females from each of 19 isofemale lines, subsequently maintained in a population cage. F_1_ daughter’s productivity was measured as both 7 day productivity and LRS (as above for parentals). Due to experimental constraints, F_1_ son’s productivity was measured as 7 day productivity; F_1_ son’s LRS was not measured.

### Data analysis

#### Multiple regressions

Additive effects can be detected by regressing offspring values on parental values [40]. To detect paternal and maternal genetic effects, crosses were grouped by sire line (across different dam lines) or dam line (across different sire lines) and regressed on values of paternal and maternal lines [41]. The model for paternal effects of LRS productivity on daughter LRS had a non-normal distribution and so a quasipoisson distribution was used to calculate pseudo R^2^; all other comparisons were normally distributed. Multiple testing was corrected using false discovery rate (FDR). Analyses were performed in R 3.0.3 [42].

#### Cockerham and Weir Biomodel

Reproductive success measures were analyzed by the Cockerham and Weir Biomodel [38, 43] which allows for an estimation of genetic (additive and non-additive), maternal and paternal variance components for reproductive success (Additional file 1: Table S1). The maternal and paternal variance components include genetic and non-genetic nuclear, cytoplasmic, and environmental effects. Data for inbred crosses (crosses either made with or resulting from dams and sires from the same isofemale line) were removed from analysis in the model as recommended. The equation of the model was

$$ {Y}_{i j kl} = \mu + {N}_i + {N}_j + {T}_{i j} + {M}_j + {P}_i + {K}_{i j} + {R}_{k(ij)} + {W}_{l\left( k(ij)\right)} $$

where Y_*ijkl*_ is the reproductive success of the *l*’th individual from the *k*’th replicate of cross between male line *i* and female line *j*, μ is the mean reproductive success of the population. N_*i*_ and N_*j*_ are the haploid nuclear additive effects of lines *i* and *j*, independent of sex. T_*ij*_ is the haploid nuclear nonadditive interaction (including dominance and epistatic effects). M_*j*_ and P_*i*_ are the maternal and paternal genetic and environmental effects of line *j* when used as dams and line *i* when used as sires. K_*ij*_ is the interaction between maternal and paternal effects. R_*k(ij)*_ is the effect of *k*’th replicate cross within dam x sire line combinations. W_*l(k(ij))*_ is the within replicate cross (the residual) effect of individual *l* [41, 44–46]*.* Note that the analysis for parentals’ reproductive success does not contain the term W_*l(k(ij))*_ as there is no within-replicate cross (residual) effect of individuals.

The Cockerham and Weir Biomodel was fitted using the GLIMMIX procedure in SAS 9.3 [47]. The EFFECT command was used to define the nuclear parental contributions as a multimember effect ([47]: Example 38.16, pg 2412). The COVTEST command was used to provide a likelihood ratio test to compare a reduced model, where a given covariance parameter is set to zero, to a full model where all parameters were allowed to have positive values.

Observational variance parameters (Additional file 1: Table S1) were used to calculate causal variance parameters using F, the inbreeding coefficient [45]. Isofemale lines are estimated to have a total inbreeding coefficient of F = 0.44. This inbreeding coefficient is estimated from F_IT_ = F_ST_ + F_IS_(1-F_ST_) [48], assuming: (1) a population bottleneck of 2 individuals and that the individual female caught from the wild used to start the isofemale line was mated to a single male or that there is strong second-male sperm precedence (drift inbreeding) and (2) a full brother and sister sibling mating in the population (pedigree inbreeding). This level of inbreeding is slightly less than that of previous studies that have used the Cockerham and Weir Biomodel, which have inbreeding coefficients of approximately 0.67-0.89 [41, 45, 46].

#### Inbred vs. Outbred

The effects of inbreeding on productivity were calculated using Linear Mixed Models (LMM). A nested LMM was used with inbred or outbred as a fixed factor and female line as the random factor. The productivity of inbred vs. outbred crosses were compared within each isofemale line for productivity; this assesses whether pairing of related gametes (producing inbred offspring) affects productivity. The productivity of inbred vs. outbred F_1_ sons and daughters were also compared; this assesses whether inbreeding (being inbred yourself) affects productivity. Total inbred and outbred values were analyzed using Welch’s test. Analyses were performed in R 3.0.3 [42].

## Results

### Comparison of productivity timescales

Productivity was measured as both the number of offspring produced in 7 days (7 day productivity) and the number of offspring produced over the entire female’s lifespan (lifetime reproductive success: LRS) for the parental combinations and for the F_1_ daughters (Additional file 2: Table S3). The regression of these two measures of productivity was previously shown to be positive and significant for parentals [39] and we find that it is also significant for daughters (Table 1; R^2^ = 0.108, d.f. = 98, *P* = 0.0008), indicating concordance between 7 day productivity and lifetime reproductive success for these groups.

**Table 1:** Pairwise correlation values (*r*) and statistical significance (*P*) for the number of offspring produced when measured as parental lifetime reproductive success (LRS), daughter LRS, parental 7 day, daughter 7 day, and son 7 day productivity; bold values are statistically significant after False Discovery Rate correction for multiple tests

	Daughter LRS		Parent 7 day		Daughter 7 day		Son 7 day	
	*r*	*P*	*r*	*P*	*r*	*P*	*r*	*P*
Parent LRS	0.3270	**0.0009**	0.513	**<0.0001** ^1^	0.248	**0.0130**	0.296	**0.0028**
Daughter LRS			0.081	0.4252	0.329	**0.0008**	0.078	0.4422
Parent 7 day					0.233	**0.0199**	0.317	**0.0013**
Daughter 7 day							0.343	**0.0005**

^1^Reproduced from [39]

**Table 2:** Observational variance component estimates from the Cockerham and Weir Biomodel to estimate the genetic architecture of lifetime reproductive success measures in isofemale lines of *D. melanogaster* and their F_1_ offspring

	F_1_ daughters LRS		Parent LRS		F_1_ sons 7d		F_1_ daughters 7d		Parent 7d	
Var	Estimate (SE)	*P* - val	Estimate (SE)	*P* - val	Estimate (SE)	*P* - val	Estimate (SE)	*P* - val	Estimate (SE)	*P* - val
σ^2^_N_	0.0025 (0.0015)	**0.0079**	0.0106 (0.0079)	0.0932	0.0002 (0.0004)	0.5273	0.0003 (0.0005)	0.5499	0.0037 (0.0029)	0.0689
σ^2^_T_	0.0008 (0.0014)	0.5499	0	-	0	-	0	-	0	-
σ^2^_M_	0	-	0.0020 (0.0067)	0.7530	0	-	0.0002 (0.0007)	**0.0004**	0.0004 (0.0027)	0.8703
σ^2^_P_	0	-	0.0069 (0.0082)	0.2955	0.0001 (0.0006)	0.8040	0.0003 (0.0007)	0.6319	0	-
σ^2^_K_	0	-	0	-	0	-	0	-	0	-
σ^2^_R_	0.0186 (0.0032)	**<0.0001**	0	-	0.0189 (0.0024)	**<0.0001**	0.0244 (0.0027)	**<0.0001**	0	-
σ^2^_W_	43.5622 (1.9144)		73.4872 (5.6284)		5.1874 (0.2254)		4.1653 (0.1844)		9.1237 (0.6944)	

### Generational comparisons of productivity

**Fig. 1:**
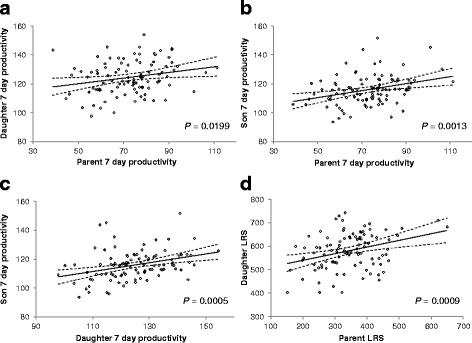
Regressions of productivity (number of offspring). Regression of **a** 7 day productivity of F_1_ daughters on parents, **b** 7 day productivity of F_1_ sons on parents, **c** 7 day productivity of F_1_ sons on F_1_ daughters, and **d** LRS productivity of F_1_ daughters on parent LRS. Dashed lines represent 95% CI

The regression of the number of offspring produced within the first week (7 day productivity) of offspring production for daughters (Table 1; Fig. 1a; R^2^ = 0.054, d.f. = 98, *P* = 0.0199) and sons (Table 1; Fig. 1b; R^2^ = 0.100, d.f. = 98, *P* = 0.0013) on parental 7 day productivity detected significant additive genetic effects. The slope of the regression gives the heritability values of productivity of sons and daughters [40]. The heritability of 7 day productivity for sons is 0.240 ± 0.072 (mean ± SE) and for daughters is 0.195 ± 0.082 (mean ± SE). There was a strong positive association between the 7 day productivity of sons and daughters (Table 1; Fig. 1c; R^2^ = 0.343, d.f. = 98, *P* = 0.0005).

The comparison of parental and daughter LRS was also significant (Table 1; Fig. 1d; R^2^ = 0.107, d.f. = 98, *P* = 0.0009), with a heritability of 0.282 ± 0.082 (mean ± SE). This comparison cannot be made for sons, as son LRS was not measured. However, there is a significant positive relationship between son 7 day and parental LRS (Table 1; R^2^ = 0.088, d.f. = 98, *P* = 0.0028).

**Fig. 2:**
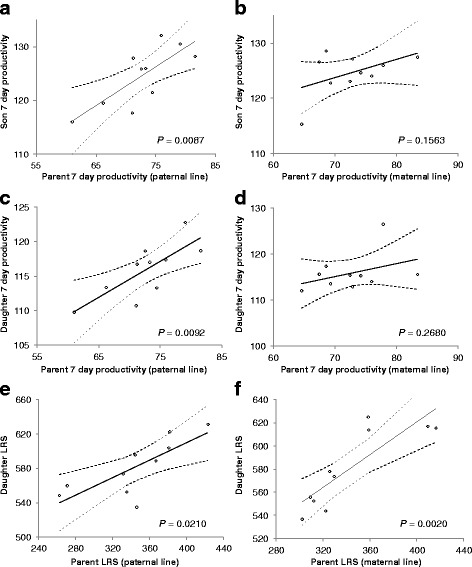
Regression of 7 day productivity of F_1_ daughters, grouped by **a** sire lines or **b** dam lines, on 7 day parental productivity detected significant paternal effects. Regression of 7 day productivity of F_1_ sons, grouped by **c** sire lines or **d** dam lines, on parental 7 day productivity detected significant paternal effects. Regression of LRS productivity of F_1_ daughters, grouped by **e** sire lines or **f** dam lines, on parental LRS productivity detected significant paternal and maternal effects. Dashed lines represent 95% CI

When the 7 day productivity data was grouped by sire or dam, we detected a significant paternal (Fig. 2a; R^2^ = 0.599, d.f. = 8, *P* = 0.0087) but not a significant maternal (Fig. 2b; R^2^ = 0.234, d.f. = 8, *P* = 0.1563) genetic effect for productivity of daughters. Similarly, we detected a significant paternal (Fig. 2c; R^2^ = 0.593, d.f. = 8, *P* = 0.0092) but not a significant maternal (Fig. 2d; R^2^ = 0.151, d.f. = 8, *P* = 0.2680) genetic effect for productivity of sons. In contrast, LRS values of daughters showed both significant paternal (Fig. 2e; pseudo R^2^ = 0.499, d.f. = 8, *P* = 0.021) and maternal (Fig. 2f; R^2^ = 0.701, d.f. = 8, *P* = 0.002) genetic effects when the data was grouped by sire or dam LRS productivity.

### Partitioning of productivity variance

The Cockerham and Weir Biomodel partitions the productivity variance into genetic and parental effects. We used isofemale lines that were not inbred in order to reduce the effect of inbreeding depression on our measures of productivity, but it should be noted that the lower inbreeding coefficient of the lines reduces the strength of the Biomodel. The model detected no significant additive or non-additive genetic effects, maternal or paternal effects (which includes genetic and non-genetic nuclear, cytoplasmic and environmental), or interaction effects for LRS or 7 day productivity of parentals or 7 day productivity of F_1_ sons (Table 2). The LRS productivity of F_1_ daughters is influenced by significant nuclear additive genetic effects (*P* = 0.0079), while the 7 day productivity of F_1_ daughters is influenced by significant maternal genetic effects (*P* = 0.0004; Table 2). However, the significant nuclear additive genetic effects accounts for only 0.03% of the variation in daughter LRS productivity, and the significant maternal effects for less than 0.01% of the variation in daughter 7 day productivity (Additional file 3: Table S2). This is not surprising since reproductive success (productivity) is an extremely variable polygenic complex trait. The majority of the variation for productivity was accounted for by replicate variance (explaining over 99% of the variation; Additional file 3: Table S2).

### Comparison of inbred vs. outbred productivity

**Fig. 3:**
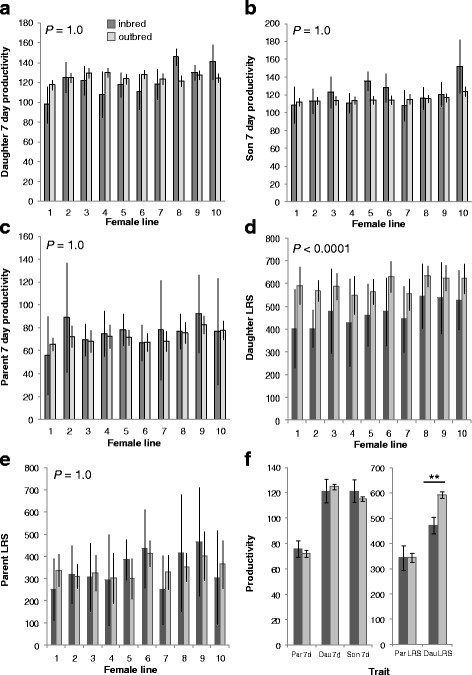
Productivity of inbred (dark grey) vs. outbred (light grey) crosses for each isofemale line. **a** 7 day productivity of F_1_ daughters, **b** 7 day productivity of F_1_ sons, **c** 7 day productivity of parentals, **d** LRS productivity of F_1_ daughters, and **e** LRS productivity of parentals. **f** Comparison of overall inbred vs. outbred values averaged across all data points. Error bars represent 95% CI. ** = *P* < 0.0001

There is no significant difference between 7 day productivity of inbred and outbred crosses of F_1_ daughters when either compared across female lines (Fig. 3a; *χ* ^2^_(1)_ = 0, *P* = 1.0) or when values are combined (Fig. 3f; t = 0.89, *P* = 0.37); the same is true for 7 day productivity of F_1_ sons (Fig. 3b and f; *χ* ^2^_(1)_ = 0, *P* = 1.0; t = 1.42, *P* = 0.19), 7 day productivity of parentals (Fig. 3C,F; *χ* ^2^_(1)_ = 0, *P* = 1.0; t = 1.02, *P* = 0.33), and lifetime reproductive success of parentals (Fig. 3e and f; *χ* ^2^_(1)_ = 0, *P* = 1.0; t = 0.04, *P* = 0.97). However, inbred crosses of F_1_ daughters have significantly lower lifetime reproductive success than outbred crosses (Fig. 3d; *χ* ^2^_(1)_ = 10.862, *P* < 0.0001), with every line that was tested showing lower productivity for inbred than outbred daughters. As expected, this comparison remains significant when the data are combined across lines (Fig. 3f; t = 5.43, *P* <0.0001).

## Discussion and conclusions

We find that parental combinations that have high productivity produce offspring with high productivity. Thus, there does not appear to be a trade-off between the direct fitness benefits of parental productivity and the indirect benefits of offspring quality, at least not for our non-competitive measures of reproductive success in this population. We also find a significant correlation between the productivity of sons and that of daughters, indicating that parents that produce highly-productive sons also produce highly-productive daughters when mated in the absence of competition. Similar positive pleiotropic effects were found between male calling effort and female fecundity in *Teleogryllus commodus* (Orthoptera: Gryllidae), indicating that good genes can be beneficial to the fitness of both males and females [49]. However, previous studies have suggested that good genes can be sex specific and detrimental to members of the opposite sex. In *Tribolium castaneum* (Coleoptera: Tenebrionidae) there was evidence of sexual conflict, where polyandrous females produced fit sons, but not fit daughters [50]. Likewise, a negative correlation was found in a laboratory population of *D. melanogaster* for adult reproductive success between females (female fecundity) and males (male ability to gain fertilizations) when placed in competition, indicating that genes conferring reproductive success to males cause a reduced fitness in females [31]. In contrast, another study found that both inbred and outbred crosses of *D. melanogaster* had no relationship between male and female fitness [51]; this discordance with the results of Chippindale et al. [31] suggests that there may be segregating genetic variation across populations. Further, our measure of productivity was in the absence of competition, and thus measured the basal ability to produce offspring, its inheritance, and response to inbreeding. This may therefore also contribute to the different results between our findings and others. It would be worth exploring within the same populations whether the components we measured produce different results in the presence of competition, indicating which productivity measures only experience selection under competitive conditions.

We found significant additive and paternal genetic effects for the 7 day productivity of F_1_ sons and both 7 day and lifetime productivity of F_1_ daughters, but only found a significant maternal genetic effect when evaluating the lifetime reproductive success of daughters; sons were not measured for this trait. We also found that F_1_ daughters had significant additive genetic effects for lifetime reproductive success and significant maternal effects for 7 day productivity when analyzed using the Cockerham and Weir Biomodel. However, unlike the regression analysis, this model did not find any other genetic or parental effects, or effects for parentals or F_1_ sons. This difference in results is likely due to the Cockerham and Weir Biomodel partitioning all of the phenotypic variation into the replicate variance, which is enhanced due to our use of isofemale lines that were not fully inbred. Similar results were found in Buzatto et al. [41], where additional regression analysis detected effects not found using the Biomodel, which they attribute to the Biomodel being conservative and underestimating the variance components. The detection of an effect in F_1_ offspring but not parentals could also be due to the larger number of replicates for this group (16 vs. 4), and the effect in lifetime reproductive success but not 7 day productivity could be due to productivity differences resulting from our different measures (ranges of 10–1220, and 3–306 offspring, respectively).

We found distinct differences among the mean productivity of parentals and F_1_ sons versus F_1_ daughters when comparing between inbred vs. outbred crosses (Fig. 3). We found that female offspring (F_1_ daughters) from inbred crosses produce significantly fewer offspring than those from outbred crosses, as we expected based on the well-known effect of inbreeding on a variety of fitness traits [43, 52, 53] and what has been reported empirically for the fitness effects of inbreeding on *D. melanogaster* reproduction in particular (e.g., [54, 55]). This indicates a cost of reduced fitness to females that are themselves inbred. Surprisingly, however, this inbreeding depression is only present in the long-term (LRS) productivity of F_1_ daughters, but not the short-term (7 day) productivity of F_1_ daughters or F_1_ sons. While it is possible that short-term reproductive success is more robust to the effects of inbreeding, laboratory strains of *D. melanogaster* have been shown to suffer reduced-short-term reproductive success [51], suggesting that the length of measurement is not the underlying reason we do not detect an effect on 7-day reproductive success. However, there are other differences in experimental design when comparing that study to ours, warranting further exploration to confirm. The presence of inbreeding effects only for lifetime reproductive success indicates that these effects are mediated by aging in daughters.

Alternatively, offspring produced from sibling matings may simply not suffer a short-term reproductive consequence if the siblings are not fully inbred themselves, as in our recently-caught lines. Supporting this argument, there was no reduction in long-term or short-term fitness for parental crosses producing inbred offspring, suggesting the absence of detectable lethality or gamete incompatibility due to mating with siblings in these recently collected lines. Short-term measures of reproductive success may be more biologically relevant than LRS in this species since, in the wild, *D. melanogaster* is predicted to have an average lifespan of approximately three days [56], although the this estimate may be low as capture-recapture methods can conflate loss due to migration with loss due to death. Further, inbred populations of *D. melanogaster* that were later outbred were able to rapidly purge deleterious alleles [57, 58], which can reduce the effect of inbreeding [59], suggesting that outbred wild-type populations have a reduced likelihood of suffering from inbreeding depression, at least in situations where the environment is relatively constant [60]. The absence of a short-term cost to inbreeding may explain why wild-type flies from this species do not avoid mating with siblings in behavior assays, and may even prefer mating with siblings [61–63], increasing their inclusive fitness [24, 64]. Thus, while females would be predicted to avoid mating with related males in order to avoid the costs of inbreeding (e.g., [21–23]), this may not be a relevant factor in wild-type populations of *D. melanogaster*.

## Additional files

Additional file 1: Table S1. Variance parameters. Table adapted from (Bilde et al. [44]; Dowling et al. [46]; Buzatto et al. [40]). (DOCX 15 kb)
Additional file 2: Table S3. Reproductive success measures of the number of offspring resulting from laying eggs. (XLSX 87 kb)
Additional file 3: Table S2. Causal variance component estimates from the Cockerham and Weir Biomodel to estimate the genetic architecture of lifetime reproductive success measures in isofemale lines of *D. melanogaster* and their F_1_ offspring. (DOCX 13 kb)
