# Supplementary material for: Cross-generational comparison of reproductive success in recently caught strains of Drosophila melanogaster
Source: BMC Evol Biol. 2017 Feb 6;17:41. doi: 10.1186/s12862-017-0887-1 (PMC5294731; doi:10.1186/s12862-017-0887-1)
Supplement: Additional file 1: Table S1. — Variance parameters. Table adapted from (Bilde et al. [44]; Dowling et al. [46]; Buzatto et al. [40]). (DOCX 15 kb) [file 12862_2017_887_MOESM1_ESM.docx]

**Supplementary Table 1.** Variance parameters. Table adapted from (Bilde et al. 2008; Dowling et al. 2010; Buzatto et al. 2012).

| Observational  variance | Causal variance ^*^ | Description |
| --- | --- | --- |
| σ^2^_N_ | V_A_ = 2 σ^2^_N_ / F | Nuclear additive variance |
| σ^2^_T_ | V_D_ = σ^2^_T_ / F^2^ | Nuclear interaction variance (dominance, if epistatic is small) |
| σ^2^_M_ | V_M =_ σ^2^_M_ | Maternal effects variance (both genotype and environmental effects) |
| σ^2^_P_ | V_P =_ σ^2^_P_ | Paternal effects variance (both genotype and environmental effects) |
| σ^2^_K_ | V_K =_ σ^2^_K_ | Interaction variance (of maternal and paternal effects and of nuclear and extra-nuclear effects) |
| σ^2^_R_ | V_E_ = σ^2^_R +_ σ^2^_W_ ^†^ | Among replicate crosses variance |
| σ^2^_W_ | V_E_ = (V_TOT_  - V_A -_ V_D -_ V_M -_ V_P -_ V_K_ ) | Within replicate crosses variance |
|  | V_TOT_ = (σ^2^_N +_ σ^2^_T +_ σ^2^_M +_ σ^2^_P +_ σ^2^_K +_ σ^2^_R +_ σ^2^_W_) |  |

^*^ F is the inbreeding coefficient.

^†^ Only used if F = 1
