# Supplementary material for: Cross-generational comparison of reproductive success in recently caught strains of Drosophila melanogaster
Source: BMC Evol Biol. 2017 Feb 6;17:41. doi: 10.1186/s12862-017-0887-1 (PMC5294731; doi:10.1186/s12862-017-0887-1)
Supplement: Additional file 3: Table S2. — Causal variance component estimates from the Cockerham and Weir Biomodel to estimate the genetic architecture of lifetime reproductive success measures in isofemale lines of D. melanogaster and their F1 offspring. (DOCX 13 kb) [file 12862_2017_887_MOESM3_ESM.docx]

**Supplementary Table 2.** Causal variance component estimates from the Cockerham and Weir Biomodel to estimate the genetic architecture of lifetime reproductive success measures in isofemale lines of *D. melanogaster* and their F_1_ offspring.

|  | **F_1_ daughters LRS** | | **Parent LRS** | | **F_1_ sons 7d** | | **F_1_ daughters 7d** | | **Parent 7d** | |
| --- | --- | --- | --- | --- | --- | --- | --- | --- | --- | --- |
| **Var** | **Estimate** | **%** | **Estimate** | **%** | **Estimate** | **%** | **Estimate** | **%** | **Estimate** | **%** |
| **V_A_** | 0.0114 | 0.03 | 0.0480 | 0.06 | 0.0009 | 0.02 | 0.0003 | <0.01 | 0.0037 | 0.04 |
| **V_D_** | 0.0036 | 0 | 0 | 0 | 0 | 0 | 0 | 0 | 0 | 0 |
| **V_M_** | 0 | 0 | 0.0019 | 0 | 0 | 0 | 0.0002 | <0.01 | 0 | 0 |
| **V_P_** | 0 | 0 | 0.0068 | 0 | 0.0001 | 0 | 0.0003 | <0.01 | 0.0004 | <0.01 |
| **V_K_** | 0 | 0 | 0 | 0 | 0 | 0 | 0 | 0 | 0 | 0 |
| **V_E_** | 43.5690 | 99.96 | 73.4497 | 99.94 | 5.2055 | 99.98 | 4.1653 | 99.98 | 9.1237 | 99.95 |
| **V_TOT_** | 43.5840 |  | 73.5064 |  | 5.2065 |  | 4.1661 |  | 9.1279 |  |
